# Deep sequencing of the tobacco mitochondrial transcriptome reveals expressed ORFs and numerous editing sites outside coding regions

**DOI:** 10.1186/1471-2164-15-31

**Published:** 2014-01-17

**Authors:** Benjamin T Grimes, Awa K Sisay, Hyrum D Carroll, A Bruce Cahoon

**Affiliations:** 1 Department of Biology, Box 60, Middle Tennessee State University, Murfreesboro, TN 37132, USA; 2 Computational Science Program, Middle Tennessee State University, Box 48, Murfreesboro, TN 37132, USA

**Keywords:** Mitochondrial transcriptome, Plant mitogenome, Tobacco, *Nicotiana tabacum*, RNA editing

## Abstract

**Background:**

The purpose of this study was to sequence and assemble the tobacco mitochondrial transcriptome and obtain a genomic-level view of steady-state RNA abundance. Plant mitochondrial genomes have a small number of protein coding genes with large and variably sized intergenic spaces. In the tobacco mitogenome these intergenic spaces contain numerous open reading frames (ORFs) with no clear function.

**Results:**

The assembled transcriptome revealed distinct monocistronic and polycistronic transcripts along with large intergenic spaces with little to no detectable RNA. Eighteen of the 117 ORFs were found to have steady-state RNA amounts above background in both deep-sequencing and qRT-PCR experiments and ten of those were found to be polysome associated. In addition, the assembled transcriptome enabled a full mitogenome screen of RNA C→U editing sites. Six hundred and thirty five potential edits were found with 557 occurring within protein-coding genes, five in tRNA genes, and 73 in non-coding regions. These sites were found in every protein-coding transcript in the tobacco mitogenome.

**Conclusion:**

These results suggest that a small number of the ORFs within the tobacco mitogenome may produce functional proteins and that RNA editing occurs in coding and non-coding regions of mitochondrial transcripts.

## Background

Angiosperm mitochondrial genomes range from 200,000 to more than 2.6 million bp. These large size differences are due to highly variable intergenic regions that lie between a relatively conserved set of protein coding genes [1,2]. Inter-species comparisons of mitogenomes suggest they undergo frequent inter- and intra-molecular recombination and tend to acquire both chloroplast and nuclear genetic material [3]. In addition, short degenerate repeats are common between genes in cucurbit mtDNA [4]. Another contributing force to the chimeric nature of plant mitochondrial genomes is their ability to readily uptake DNA through horizontal gene transfer. Richardson and Palmer [5] showed that the mitochondria of the dicot *Amborella trichopoda* contained sequences homologous to different species’ mitogenomes. With their highly recombinant DNA, propensity for genomic double strand breakage, and perpetual ability to undergo fusion and fission, these organelles set themselves apart from the rest of the cell regarding potential for genomic diversity [2].

The frequent recombination and transfer events have not only expanded the intergenic regions, but also produce possible protein-coding open reading frames (ORFs) in some species. Small ORFs can comprise a significant amount of the mitogenome; for example there are 117 poorly characterized small ORFs in the tobacco mitogenome, compared to 60 genes with identifiable functions [6]. Almost all of these are uncharacterized in tobacco, but some homologous sequences have been linked to cytoplasmic male sterility (CMS) in other species [7,8]. *Orfs 25* and *265* in sorghum have been shown to control CMS [9] and are conserved among the mitogenomes of *Oryza* and *Triticum*[10,11]. These mitogenomic ORFs may also indirectly modulate RNA steady-state levels since cytoplasmic message background affects RNA degradation [7].

The plant mitogenome is transcribed by phage-type (T7 and T3-like) nuclear-encoded RNA polymerase (RNAP) [12]. In eudicots, two RNAPs localize to mitochondria: RpoTm, which exclusively localizes to the mitochondria, and RpoTmp, which localizes to both mitochondria and plastids [13]. RpoTm is probably the primary polymerase and RpoTmp transcribes mitochondrial genes early in development [14]. Plant mitochondrial genes often possess multiple promoters consisting of core tetranucleotides CRTA, ATTA, and RGTA that are part of a nonanucleotide conserved sequence, CRTAaGaGA, and an AT-rich region upstream from the start site [15,16]. As more mitogenomic data has become available, genes without obvious promoter motifs and possible promoter sequences within intergenic regions have been discovered. There are two different descriptions of plant mitochondrial transcription that are linked to polymerase type. Kuhn et al. [17] have shown that RpoTmp is gene specific rather than promoter specific. This opens the possibility of *cis*-acting elements specifically directing transcription. Other studies have observed non-specific transcription of the intergenic regions resulting in large quantities of “junk” transcripts [18-20]. This finding, coupled with an observation of loosely controlled transcription termination—which produces long run-on RNAs [18]—suggests indiscriminate low-scale expression of much of the mitogenome, most likely by the RpoTm polymerase [21,22].

These long transcripts undergo a series of processing events to produce functional transcripts [23,24]. Transcript editing is a ubiquitous and widely encountered processing event in plant mitochondria. Every protein-coding sequence in a mitogenome is likely to be edited; overall editing in angiosperms are estimated to occur at about 500 sites per genome [25] with a range from 189 in *Silene noctiflora*[26], to 600 in date palm [20]. Almost all mitochondrial editing is performed through the process of cytosine to uracil conversion (C→U) [27]. Editing has been linked to generating start and stop codons, enabling protein function by altering amino acid content, and restoring fertility in cases of cytoplasmic male sterility (CMS) [28-30]. Lu and Hanson [31] demonstrated that protein products from the *atp6* gene in *Petunia* were made exclusively from completely edited transcripts within the mitochondria. Alternately, polypeptides from unedited or partially edited transcripts accumulate in *Zea mays*[32]. The consequences of this are still under investigation, but from a gene regulation perspective, partially edited transcripts can potentially provide a variety of gene products from a single coding region [25].

In this study, deep sequencing was used to assemble the tobacco mitochondrial transcriptome. This enabled the determination of mono- and polycistronic transcripts, identification of expressed uncharacterized ORFs, and a whole transcriptome level estimate of editing sites. We found nine monocistronic and sixteen polycistronic transcripts. Eighteen uncharacterized ORFs were transcribed, eleven of which were found to be polysome associated. Six hundred and thirty five potential edits were found with 562 occurring within protein coding genes.

## Results

### Deep sequencing and alignment of the tobacco mitochondrial transcriptome

**Figure 1:**
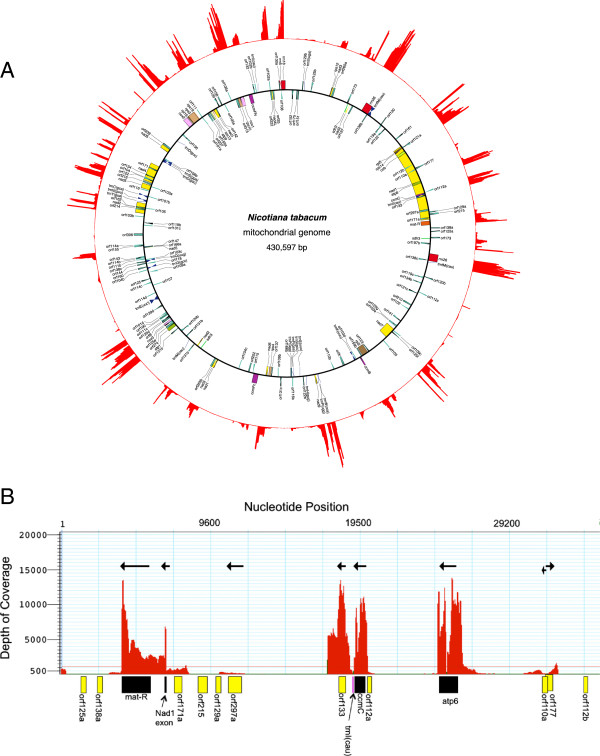
**The Tobacco Mitochondrial Transcriptome. A** – Illustration of transcript depth of coverage for the *Nicotiana tabacum* mitogenome. The figure was generated using abundance data from Lasergene’s SeqMan Pro v. 3 (DNASTAR, Madison, WI, USA) which were converted to circular coordinates using a custom perl script and drawn by gnuplot ( http://www.gnuplot.info). The inner circle shows the location of the genes and was generated from GenBank accession NC_006581 using Organellar Genome DRAW [33]. **B** – Higher-resolution view of the first 35,000 bp of the mitogenome. The depth-of-coverage chart was generated using Lasergene’s Seqman Pro v. 3. Protein-coding genes (black boxes) and open reading frames (ORFs, yellow boxes) were manually placed below each area based on a finer nucleotide map. Arrows represent the predicted transcription direction for each transcribed area.

Total RNA from tobacco leaves collected from six plants was sequenced in a single Illumina run and aligned to the tobacco mitochondrial genome as deposited in GenBank (NC_006581.1 and [6]), including repeated regions. 4,539,709 reads with an average length of 100nt aligned to the mitogenome and the resulting depth of coverage (DOC) chart revealed discrete regions with moderate to high DOC separated by spans of very low to non-existent coverage (Figures 1A & B, Additional file 1: Figure S1). The low coverage regions made up the majority of the mitogenome, with 57.2% having a DOC below 150, 51.3% below 100, and 36% below 50. The areas with the highest depth of coverage (>1000) were associated with protein-coding regions and ribosomal RNA genes despite having purposefully reduced rRNA content as part of the sequencing library preparation (see materials and methods). Four high DOC areas with no apparent coding regions were also observed - 46,685-47,000, 177,020-178,060, 337,770 - 340,520 and an area containing orf101d and orf111c from base 254,600 - 256,300. All have homologous regions in the chloroplast genome and the high DOC very likely represented alignment of both mitochondrial and chloroplast transcripts since total RNA was sequenced. tRNAs generally had low yet variable DOC’s ranging from 13 to 544 (Additional file 2: Table S1).

The alignment of protein-coding regions with the DOC chart suggests nine are produced as monocistronic transcripts (Table 1). These include six complete coding regions (*ccmC, atp6, nad9, orfx, ccmB*, and *rps12*) and three exons (nad1_exon1, nad2_exon2 and nad5_exon3). The remaining coding regions appear to be transcribed as polycistronic units.

**Table 1:** Poly- and mono-cistronic transcripts as predicted from the tobacco mitogenome transcriptome assembly

**Area**	**Strand**	**Transcript**
Sequence 1	-	matR::nad1_ex4
	+	ccmC
	-	atp6
	-	cob::rps14::rpl5::nad1_ex5
Sequence 2	+	nad1_ex1
	+	ccmFc_ex1::ccmFc_ex2
	-	nad6::rps4
	+	nad9
	-	tatC(orfx)
	+	ccmB
	-	orf159b::rpl2_ex1::rpl2_ex2
	-	nad7_ex1::nad7_ex2::nad7_ex3::nad7_ex4
Sequence 3	+	rps12
		rrn18
		rrn5
	-	orf25(atp4)::nad4L
	-	ccmFN::cox1::rps10_ex1::rps10_ex2
	-	nad1_ex3::nad1_ex2::rsp13::atp9
	+	rps19::rps3_ex1::rps3_ex2::rpl16::cox2_ex1::cox2_ex2
	+	nad5_ex4::nad5_ex5
	+	nad4_ex1::nad4_ex2::nad4_ex3::nad4_ex4::nad5_ex1::nad5_ex2
	+	nad2_ex3::nad2_ex4::nad2_ex5
	-	nad5_ex3
	+	atp8::cox3::atp1
	-	nad2_ex2
Repeat 1		rrn26
	-	orf197
Repeat 2	-	nad2_ex1::sdh3
Repeat 3	+	orf265::nad3

Two genes, *cox1* and *atp6*, exhibited a precipitous drop of DOC in the middle of these coding regions. These were considered possible uncharacterized transcript processing events so end-point PCR and RT-PCR were performed on DNA and RNA, respectively. Both PCR and RT-PCR reactions yielded amplicons of equal size, consistent with the published genome annotation (data not shown). This suggests the low DOC in these two genes does not indicate a processing event but is instead a technical inconsistency

### RNA edit sites

Potential C→U edit sites were identified in the transcriptome assembly by comparing RNA reads to the published mitogenome sequences (GenBank accessions NC_006581.1 and BA000042). Edit sites were chosen if the DOC was >200 and the RNA edit percentage less than 100%; nucleotides with a 100% change rate between RNA and the genome sequence were considered SNPs. This methodology identified 540C→U edit sites across the entire mitogenome (Additional file 3: Table S2). When compared to previously identified edit sites (PIES), this methodology failed to recognize 95 PIES but found 119 potential new sites. Combined, PIES and new sites equaled 635 total edit sites. A supermajority of the sites, 573, were in protein-coding regions and included every identifiable protein-encoding transcript plus five transcribed orfs. Only two exons, rpl2_exon1 and rps3_exon1, did not have potential edits. Among the 119 newly identified edit sites, 41 were found in coding regions, 5 in tRNAs, and 73 in intergenic regions. Forty three of the intergenic edits were in 5′ and 3′ UTRs, 23 in intergenic regions of polycistronic transcripts, and 7 in regions that were not coding regions or linked to any identifiable transcript (Additional file 4: Table S3).

### ORF steady state RNA abundance analysis

There are 117 predicted but uncharacterized ORFs in the tobacco mitogenome annotation [6]. All uncharacterized ORFs in the published mitogenome annotation were compared to the DOC chart generated in this study and a number of them occurred in regions where DOC was above background. Since transcription could signify importance, all ORF’s with a DOC >200 that did not overlap an identifiable protein-coding region were chosen for further analysis (Table 2).

**Table 2:** List of open reading frames chosen for this study

**ORF**	**Genome start site**	**Genome stop site**	**Peak DOC**	**Number of edit sites**	**Polysome association**		**Protein homologies of Polysome associated ORFs**
					**Super**	**Pellet**	
Cox2(+Control)	156352	158494	17900	14	+	+	
Cox2+EDTA					+	-	
Background	48550	49035	<75	-	+	-	
177	31683	32216	1537	-	+	+	none found
197	74704	75297	1900	5	+	-	
265/atp8	85475	86272	2481	2	+	+	full length ATPase subunit 8
129b	100135	100524	386	-	+	-	
175	102481	103008	215	-	-	-	
25/atp4	113853	114449	13360	10	+	+	full length ATPase subunit 4
222	114996	115664	8288	-	+	+	none found
239	171890	172609	1194	-	+	-	
216	191161	191811	5943	-	+	+	fragment of ribosomal protein S1
306	215367	216287	365	-	+	-	
147	221540	221983	6105	-	+	+	full length ribosomal protein L10
144	229441	229875	311	-	+	-	
118	229726	230082	387	-	+	-	
160	231140	231622	816	-	+	+	none found
125d	257199	257576	4404	-	+	-	
115	306250	306597	424	-	+	+	none found
166b	324805	325305	1182	-	+	+	fragment of cytochrome coxdase
159b/rpl10	360283	360762	8330	4	+	+	full length ribosomal protein L10

Location of start and stop codons refer to GenBank accession NC_006581.1. Peak DOC refers to the approximate highest depth of coverage noted for that transcript. Edit sites are the number of possible C→U edits found within the coding regions.

Deep-sequencing results were confirmed with qRT-PCR analysis of three biological replicates (two technical replicates from each biological for a total of *n* = 6) and Mann–Whitney non-parametric analysis was used to determine significant differences. For all qRT-PCR experiments, the *cox2* mitochondrial protein-coding gene was used as a positive control and for normalization. Background was measured from the orf161 region, which had a DOC below 75 and qRT-PCR copy number estimates well below transcribed regions.

**Figure 2:**
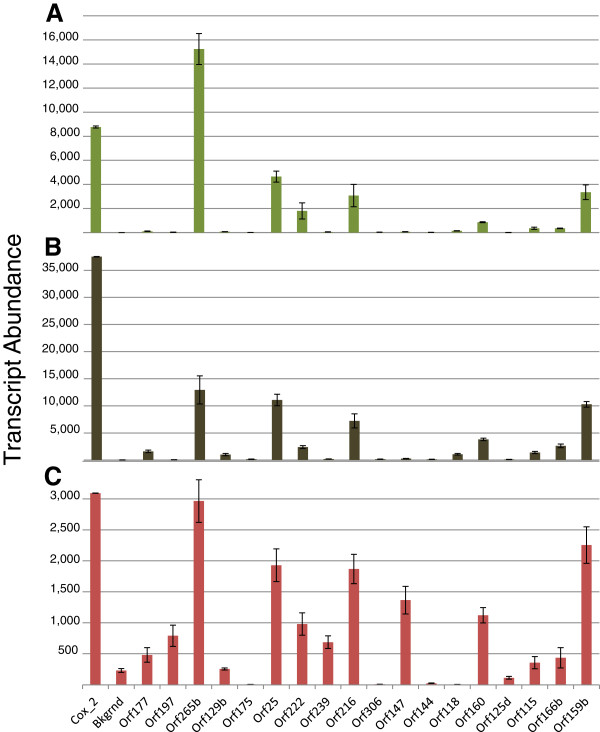
**qRT-PCR Analysis of Tobacco Mitogenome ORF Transcript Abundance.** Total RNA was extracted from three biological replicates of leaf **(A)**, root **(B)**, and flower **(C)** tissues and quantified using reverse transcriptase quantitative PCR. Transcript abundance was calculated using a derivation of the methodology of Alvarez et al. [34]. All values were normalized to *cox2* abundance as a control. DNA contamination was estimated by RT minus controls and calculated values were subtracted from the measured transcript abundance.

Leaf, root, and whole-flower RNA samples were used to compare and contrast ORF expression. qRT-PCR results suggested that steady-state levels of the 18 open reading frame transcripts were highest in roots, followed by leaves, then flowers (Figure 2 and Additional file 5: Table S4). In leaves and roots, all ORF transcripts were present at levels above background, which confirmed the RNA-seq results. In flowers, only 10 of the ORFs were significantly higher than the measured background. The most abundant ORF transcripts in all three organs were *265, 25, 222, 216, 160, 166*, and *159*.

### Polysome analysis of open reading frames

All the confirmed transcribed ORFs were subjected to polysomal analysis to test for evidence of translation. *Cox2* was used as a positive control and *orf161* to measure background. *Cox2* was also quantified in EDTA-treated extracts as a negative control. RNA in polysomal pellets and supernatants from three biological replicates were purified and measured by qRT-PCR and Mann–Whitney non-parametric analysis was used to determine significant differences. *Orfs177, 265/atp8, 25/atp4, 222, 216, 147, 160, 115, 166b*, and *159b/rpl10* were found in polysomal pellets at significantly higher amounts than background (Table 2 and Additional file 6: Table S5). All but one (*orf175*) was successfully detected in the supernatant at levels significantly higher than background.

### ORF homologies

Translations of the eleven polysome-associated ORFs were screened using GenBank ( http://www.ncbi.nlm.nih.gov) and Sol Genomics Network ( http://solgenomics.net) to see if any encode identifiable proteins found in other mitochondrial genomes. *Orfs 147* and *159b* encode putative full-length RPL10 proteins, *orf216* encodes a full-length mitochondrial rps1, *orf25* encodes a full-length *atp4* coding region, and o*rf265b is atp8. Orfs160, 115*, and *166b* do not encode identifiable proteins. *Orfs177* and *222* match uncharacterized nuclear genes from *Nicotiana benthamiana*. Ten of the ORFs were found in the mitochondrial genomes of other genera; only *orf222* was unique to *Nicotiana*.

## Discussion

### The tobacco mitochondrial transcriptome

Deep sequencing of the tobacco mitochondrial transcriptome detected multiple mono- and polycistronic transcripts with relatively long 3′ and 5′ UTRs. The most highly transcribed regions contained protein-coding regions, and the length and content of the transcripts suggested extensive post-transcriptional processing takes place. It has been suggested that mitochondrial genes between 3,000 and 8,000 nucleotides apart will most likely be transcribed as a cluster [21] which are then processed and edited to form translatable mRNAs. Pre-mRNA maturation takes place in the matrix with 5′ end modification occurring through endonuclease activity or, at times, the pre-mRNA strand does not need 5′ processing; the 5′ end is simply the beginning of the reading frame [35]. The 3′ processing is much more nebulous in nature, whereas some of the transcripts have been proposed to be terminated with the help of mitochondrial transcription termination factors (mTERFs), other strands simply run on well past the end of the reading frame— the latter situation suggests that transcription termination in plant mitochondria is not critically important [36,37]. Nevertheless, the 3′ end is still important in protecting the RNA from exonuclease activity, as local cis-acting elements aid in step loop formation [38].

In our analysis, intron and exon steady state abundance were generally distinguishable with exonic DOC much higher than intronic, suggesting that introns are degraded after removal. The intergenic regions were mostly non/low-transcribed, although there were some regions with high DOC. Some of these had ORFs predicted in the initial tobacco mitogenome annotation.

Our observations confirm that long mitochondrial transcripts are produced, but these transcripts primarily contained protein-coding regions. More than 57% of the transcriptome had a DOC below 150 and protein-coding regions were clearly distinguishable from intergenic spaces. This suggests transcription was focused on protein-coding regions and that transcription is only occurring at high rates in specific areas. Plant mitochondrial gene expression is portrayed as a relaxed and inefficient coordination of two phage-type RNA polymerases (RpoTm and RpoTmp) [17]. This is based on the placement of promoters that have been found upstream of protein coding genes as well as scattered throughout intergenic regions [17]. The overall result is long run-on transcripts found throughout plant mitochondrial transcriptomes and large quantities of cryptic transcripts from intergenic regions [36,37]. Alternatively, mitochondrial transcription rates have been shown to vary considerably between different genes – most likely due to different promoter strengths and unique stoichiometric needs of each gene [7].

### Mitogenome edit sites

Transcriptome analysis revealed 540C→U edits in the tobacco mitochondrial transcriptome which, if combined with PIES, gives 635 total edit sites with 562 in coding regions. This observation is consistent with estimates from other higher plant mitochondria, where an average of 500 edit sites per mitogenome has been suggested, with most of those edits occurring in coding regions [25]. In our analysis, transcriptome assembly accompanied by the SNP function in the assembly package failed to recognize 95 of the previously reported sites but identified 119 edit sites unreported in previous analyses (GenBank accession BA000042 and [39]).

The sites missed in the transcriptome analysis were manually inspected and all appear to be edited according to the alignment, although some at very low rates (<10%). We suspect these sites were not identified by the SNP function because they were in areas with erroneously low mid-transcript DOC’s and/or the percentage of edited nucleotides was much lower than expected based on other sites (<50%).

Most of the 119 newly identified edit sites were not in coding regions revealing a larger proportion of intergenic edit sites than identified in previous screens. Very few instances of editing in non-coding regions exist. In *Arabidopsis thaliana* 15 of 456 sites are reported outside of coding regions [40] and in tobacco, one site had been identified (NC_006581.1 and [6]). A definitive reason to edit non-coding regions is elusive, but some have been linked to splicing [41,42]. Most of the non-coding region editing sites found in this study were in UTR regions suggesting they may be necessary for processing or translation. Others found in intron regions could be a prerequisite for splicing. It is also possible that some of these are unnecessary and indicate superfluous editing similar to the silent editing reported in some third codon positions [43].

### Expression of mitogenomic open reading frames

In this study, 18 uncharacterized open reading frames were found in transcribed regions and then confirmed with qRT-PCR. Polysome analysis showed 10 transcripts from those ORF’s were attached to ribosomes. Two, *orfs25* and *265b* are homologous to *atp4* and *8*, have been linked to cytoplasmic male sterility in sorghum [9], and are found in at least two other mitogenomes [10,11]. Three others encode putative full-length proteins. Orf159b was recently characterized as *rpl10* in angiosperm mitochondria, including *N. tabacum*[44]. *Orf147* also appears to encode a near full length RPL10 protein. The role of *orf147* as a second truncated *rpl10* is unknown. *Orf216* encodes a mitochondrial RPS1 protein. RPS1 has not been defined in tobacco, but has been identified in the mitogenome other plants such as wheat and primrose [45,46]. Five ORFS —*160, 115*, *166b, 177*, and *222* do not encode identifiable proteins but are transcribed and polysome associated.

There are conflicting hypotheses regarding the possible benefit mitochondrial open reading frames provide. Some suggest their origin through recombination events create a burden on the organelle’s RNA processing systems [47] which would suggest selection against their presence. Beaudet et al. [48] found some mitogenomic ORFs were mobile elements specializing in horizontal gene transfer; these were responsible for making chimeric versions of existing mitochondrial genes suggesting they benefit the organelle. Our data suggests they are not merely present but some are also transcribed and possibly translated.

## Conclusions

Transcriptome analysis of the tobacco mitogenome demonstrated that the chromosome is transcribed as discrete mono- and poly-cistronic transcripts with low- or non-transcribed intervening sequences. This confirms previous observations and suggestions of multiple promoter sites throughout the mitogenome. An inventory of RNA edit sites shows that widespread editing is not limited to coding regions in the tobacco mito-transcriptome. This suggests editing enzymes do not discriminate between coding and non-coding RNA. Some plant mitochondrial genomes have numerous uncharacterized ORFs that may be functional genes or recombinational remnants. The data presented in this study show that 18 of the 117 poorly characterized ORFs in the tobacco mitogenome are transcribed and 10 are polysome associated. This suggests that some of these produce functional proteins.

## Methods

### Plant material and RNA extraction

*Nicotiana tabacum*, var. petit Havana was used for all experiments. For leaf tissue, 2–3 cm specimens were removed from plants grown in commercial potting soil in a Percival PGC-10 incubator set for a 16 hour day/8 hour night cycle at 28°C. Root tissues were dissected from 3 three-week- old seedlings grown from surface sterilized seeds. Briefly, tobacco seeds were sterilized by washing briefly with 70% ethanol for 30 seconds followed by a 50% bleach solution for 15 minutes with agitation. Seeds were rinsed three times with sterile water and transferred to sterile Magenta boxes. Boxes were prepared by placing several layers of 2′x 2′ paper towel squares wetted with a 1/3X concentration of Miracle Gro liquid fertilizer in the bottom and autoclaving. Root tissue was excised from healthy tobacco plants using a surgical blade and dissection microscope. Flowers were removed from the same set of plants that were the source of leaf tissue. They were collected when the corolla was fully open and shedding pollen.

For RNA extraction, tissues were frozen in liquid nitrogen and ground into a powder using a mortar and pestle. RNA was extracted with Qiagen’s RNeasy kit with the additional DNAse steps (Hercules, CA) following the manufacturer’s instructions. RNA concentrations were measured using a NanoDrop Lite (Thermo Scientific, Waltham, MA).

### Deep sequencing of the tobacco mitochondrial transcriptome

Total tobacco RNA was prepared at MTSU and sent to the University of Illinois sequencing center (Springfield, IL) for transcriptome sequencing. Ribosomal RNAs were removed from the total RNA using Ribo-Zero (Epicentre, Madison, WI) and a total RNA library prepared using Illumina’s TruSeq RNAseq Sample Prep kit (San Diego, CA). Libraries were sequenced on one lane for 100 cycles from each end on an Illumia HiSeq2000 platform using a TruSeq SBS sequencing kit v.3 and analyzed with Casava 1.8. 163,836,382 sequence reads were reported with an average length of 100nt. Once sequence data was received by the team at MTSU, sequences were aligned to the tobacco mitochondrial genome (Genbank NC_006581) using DNAstar’s (Madison, WI) Seqman NGen program to produce a depth-of-coverage map.

### Polysomal RNA isolation and purification for translation analysis

Polysomal RNA was extracted and isolated using a protocol modified from Mayfield et al. [49]. 200–400 mg of leaf tissue was harvested and ground into a powder with liquid nitrogen. The frozen powder was quickly re-suspended in extraction buffer (200 mM Tris HCL, pH 9.0, 200 mM KCL, 35 mM MgCl2, 25 mM EGTA, 200 mM sucrose, 1% Triton-X 100, 2% BRIJ, 0.5 mg/ml heparin, 0.7% 2-mercaptoethanol, and 100 mg/ml chloramphenicol). Cell debris was removed by centrifugation at 17,000xg for 10 min at 4°C, supernatant transferred to a new tube, and deoxycholate added to a final concentration of 0.05%. Samples were centrifuged at 13,000xg for 10 min at 4°C. The remaining supernatant was then layered onto a two-step sucrose gradient (1.75 M and 0.5 M sucrose in 1x cushion buffer: 40 mM Tris HCL, pH 9.0, 200 mM KCL, 30 mM MgCL2, 5 mM EGTA, 0.5 mg/ml heparin, 0.7% 2-mercaptoethanol, and 100 mg/ml chloramphenicol). Polysomal RNA was pelleted by ultracentrifugation at 180,000xg for 120 min at 4°C. After ultracentrifugation, the supernatant was removed by carefully pipetting and transferring the top layers to new centrifuge tubes on ice. The polysomal RNA was extracted from pellets with Qiagen’s Plant RNeasy Kit following the manufacturer’s protocol. The nucleic acid remaining in the supernatant was precipitated by adding 4 volumes of 95% ethanol, 1/10 volume of sodium acetate, flash frozen in liquid nitrogen, thawed on ice, and pelleted by centrifugation at 30,000xg for 30 min at 4°C. All nucleic acid from polysome analyses was re-precipitated with 3 volumes of 8 M LiCl to remove residual heparin that carried through the extraction process and would inhibit reverse transcriptase [50].

As a negative control, RNA extracts were incubated with 0.5 M EDTA, vortexed, and placed on ice for 10 min to release ribosomes from transcripts. After incubation on ice, the EDTA/extract mixture was layered on top of sucrose gradients, ultracentrifuged, and processed as described above.

### Primer design

All primer pairs were prepared as described in Sharpe et al. [51]. Briefly, optimal annealing temperatures and PCR efficiencies were empirically determined using a BioRad CFX96 C1000 thermal cycler (Hercules, CA). To ensure the amplification of a single product, melt curves were inspected for each reaction and products of initial reactions were visualized on a 3% agarose gel stained with ethidium bromide. Experimental primers for qRT-PCR were designed and tested for 28 open reading frames, a positive control (*cox2*), and negative/background control (Orf161) (Additional file 7: Table S6). Primers were purchased from Eurofins MWG Operon, Inc. (Huntsville, AL). Optimal annealing temperatures, primer pair efficiencies, and amplicon lengths can be found in Additional file 7: Table S6.

### qRT-PCR

qRT-PCR analysis was performed using a Bio-Rad CFX96 C1000 Thermal Cycler (Hercules, CA). PCR reactions were prepared with 5 μl of Quanta PerfeCTa One-Step SYBR Green Mix (Gaithersburg, MD), 2ul 5pmol primers, 50 ng RNA template, 0.1 μl M-MLV reverse transcriptase (Promega, Madison, WI), and nuclease-free water for a 10 μl total reaction volume. Cycle programming started with a 30 min reverse transcriptase step at 45° followed by a 3 min 95° step. The PCR cycle stage was 95°C for 30 Sec 59° for 15 sec and 72°C for 15 sec for 39 cycles. A melt curve was included to ensure the production of single amplicons.

### Calculation of estimated copy numbers

The initial qRT-PCR runs were performed to confirm transcription shown by mitochondrial transcriptome deep sequencing. Three biological replicates were used for each tissue sample (roots, leaves, and flowers) with two technical replicates for each biological replicate. Crossover threshold values (C_t_) were used to determine estimated initial RNA amounts using a variation of the method developed by Alvarez et al. [34].

$$N_{0}=\frac{F_{t}{\left(1+E\right)}^{-C_{t}}}{\mathrm{Amp}}$$

Formula 1. *N*_*0*_ = initial amount of mRNA, *F*_*t*_ = fluorescence, *E* = reaction efficiency, *C*_*t*_ = crossover threshold, *Amp* = amplicon size.

### Statistical analysis

All copy number estimates were normalized against the lowest value of the six *cox2* positive control replicates. The normalization factor was calculated by dividing the *cox2* copy number estimate from each sample by the smallest *cox2* value. This normalization factor was then used to adjust the matching experimental biological and technical replicates across the entire data set. The normalized values were then used to apply standard descriptive statistics such as mean, median, mode, standard deviation, and standard error. The Mann–Whitney rank sum test was used to determine p-values from the normalized copy number data.

### Availability of supporting data

The Illumina transcriptome data sets supporting the results presented in this article are available in the National Center for Biotechnology Information – USA ( http://www.ncbi.nlm.nih.gov) Sequence Read Archive, accession SRX403934.

## Competing interests

The authors declare they have no competing interests.

## Authors’ contributions

BTG completed the orf analysis by identifying potentially expressed regions. He performed all qRT-PCR experiments and polysome analysis. He also wrote the majority of the manuscript which is included in his graduate thesis. AKS & HDC were responsible for the initial handling of the transcriptome data set including formatting and some analysis. They also prepared Figure 1A and wrote appropriate portions of the manuscript. HDC edited the manuscript. ABC is the PI and is responsible for the inception and execution of the project as a whole. He completed the RNA extractions and quality control measures for transcriptome sequencing and was responsible for some aspects of the assembly and analysis of the transcriptome. He wrote portions of the manuscripts and edited the whole. All authors read and approved the final manuscript.

## Supplementary Material

Additional file 1: Figure S1 A depth-of-coverage chart generated by Lasergene’s Seqman Pro v. 3. Protein coding genes (black boxes), open reading frames (ORFs, yellow boxes), ribosomal RNAs (orange boxes), and tRNAs (purple lines) were manually placed below each area based on a finer nucleotide map available through the Seqman Pro software package. All protein-coding genes and ORFs are labeled. Click here for file

Additional file 2: Table S1 All predicted tRNAs, their positions, and peak depth of coverage. Click here for file

Additional file 3: Table S2 List of the 570 potential C→U edit sites predicted from the transcriptome assembly. An initial list of possible edit sites was generated using the SNP detection function in Lasergene’s Seqman Pro v.3. The list was refined by removing all non C→U transitions, sites with a depth of coverage <200, and sites showing a 100% edit rate between the genome sequence and the transcriptome. Edit sites in protein-coding regions and selected ORFs are denoted by a bold outline with the name of the gene/ORF to the left. Edit sites in non-coding regions are not outlined. Previously identified edit sites (PIES) from Genbank accession BA000042, were included for comparison. Grey highlighted PIES were overlooked as edit sites in this transcriptome analysis and identification criteria. Click here for file

Additional file 4: Table S3 Summary of edit sites in non-coding regions and location of nucleotide in relation to the mono- and poly-cistronic transcripts. Click here for file

Additional file 5: Table S4 ORF Average Transcript Abundance and Standard Error (S.E.) as Measured by qRT-PCR and Mann–Whitney Pair-Wise Statistical Analysis. Click here for file

Additional file 6: Table S5 ORF Average Transcript Abundance and Standard Error (S.E.) in the Supernatant and Pellet Portions of Polysome Analyses as Measured by qRT-PCR and Mann–Whitney Pair-Wise Statistical Analysis. Click here for file

Additional file 7: Table S6 Primers used in this study. Click here for file
