# Supplementary figures and images for: Deep sequencing of the tobacco mitochondrial transcriptome reveals expressed ORFs and numerous editing sites outside coding regions
Source: BMC Genomics. 2014 Jan 17;15:31. doi: 10.1186/1471-2164-15-31 (PMC3898247; doi:10.1186/1471-2164-15-31)

Supplemental Figure 1

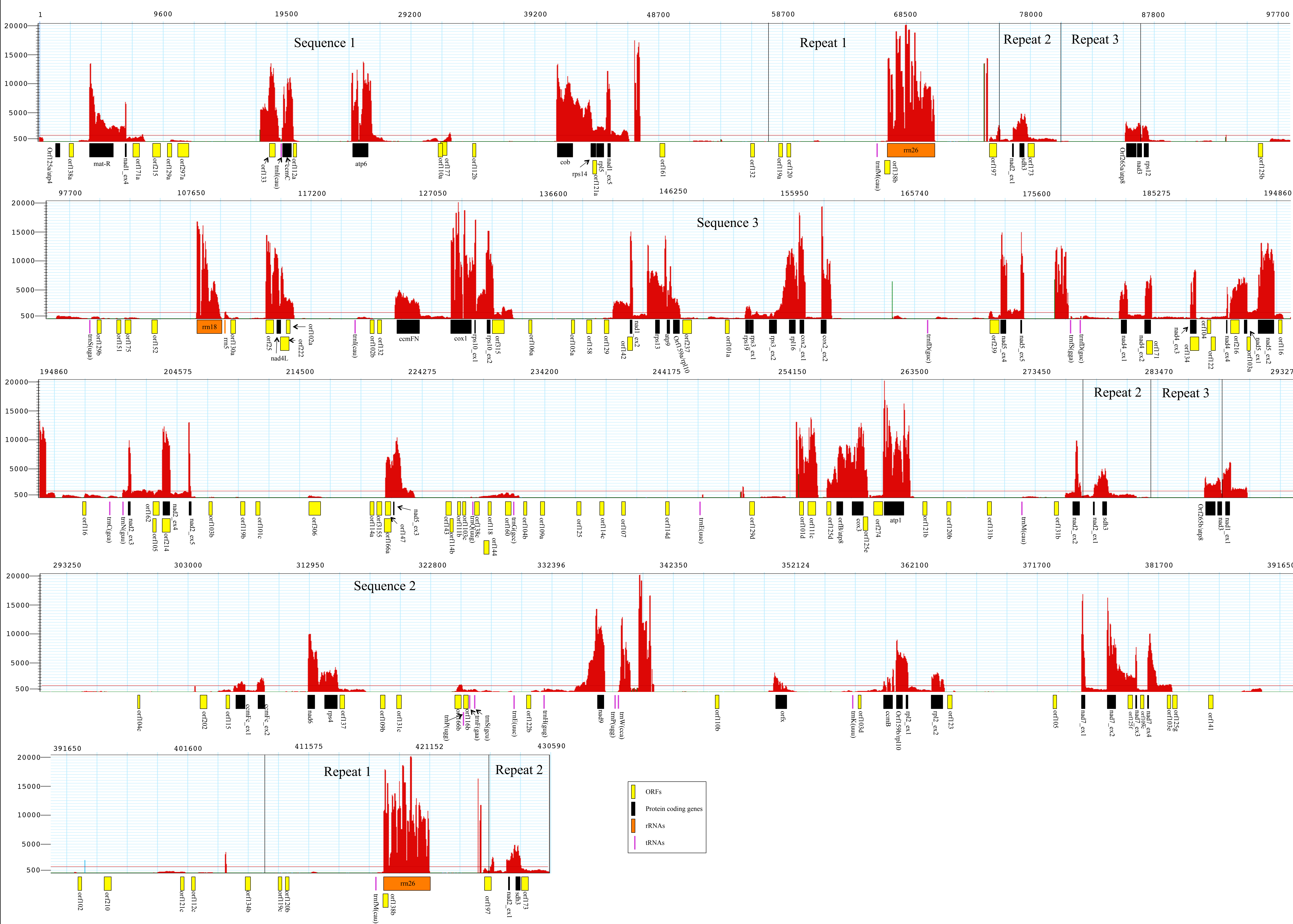

Supplement: Additional file 1: Figure S1 — A depth-of-coverage chart generated by Lasergene’s Seqman Pro v. 3. Protein coding genes (black boxes), open reading frames (ORFs, yellow boxes), ribosomal RNAs (orange boxes), and tRNAs (purple lines) were manually placed below each area based on a finer nucleotide map available through the Seqman Pro software package. All protein-coding genes and ORFs are labeled. [file 1471-2164-15-31-S1.pdf]
