# Supplementary material for: Deep sequencing of the tobacco mitochondrial transcriptome reveals expressed ORFs and numerous editing sites outside coding regions
Source: BMC Genomics. 2014 Jan 17;15:31. doi: 10.1186/1471-2164-15-31 (PMC3898247; doi:10.1186/1471-2164-15-31)
Supplement: Additional file 2: Table S1 — All predicted tRNAs, their positions, and peak depth of coverage. [file 1471-2164-15-31-S2.pdf]

Supplemental Table 1 - tRNA Depth of Coverage

tRNA	position	peak DOC
trnI(cau)	18954..19027	285
trnfM(cau)	66201..66274	45
trnS(uga)	99558..99644	59
trnI(cau)	121049..121122	55
pseudo-trnD(guc)	166843..166908	87
trnS(gga)	178217..178303	446
trnD(guc)	179000..179073	459
trnC(gca)	197450..197520	96
trnN(guu)	199441..199512	189
trnY(gua)	200481..200563	91
trnQ(uug)	228472..228543	14
trnG(gcc)	231805..231876	282
trnE(uuc)	246982..247053	490
trnM(cau)	272515..272587	160
trnP(ugg)	325367..325441	352
trnF(gaa)	325701..325774	223
trnS(gcu)	326148..326235	421
trnE(uuc)	329387..329458	20
trnH(gug)	331955..332028	557
trnP(ugg)	337551..337624	39
trnW(cca)	337782..337855	308
trnK(uuu)	356762..356834	60
trnfM(cau)	416951..417024	44
