# Supplementary material for: Deep sequencing of the tobacco mitochondrial transcriptome reveals expressed ORFs and numerous editing sites outside coding regions
Source: BMC Genomics. 2014 Jan 17;15:31. doi: 10.1186/1471-2164-15-31 (PMC3898247; doi:10.1186/1471-2164-15-31)
Supplement: Additional file 3: Table S2 — List of the 570 potential C→U edit sites predicted from the transcriptome assembly. An initial list of possible edit sites was generated using the SNP detection function in Lasergene’s Seqman Pro v.3. The list was refined by removing all non C→U transitions, sites with a depth of coverage <200, and sites showing a 100% edit rate between the genome sequence and the transcriptome. Edit sites in protein-coding regions and selected ORFs are denoted by a bold outline with the name of the gene/ORF to the left. Edit sites in non-coding regions are not outlined. Previously identified edit sites (PIES) from Genbank accession BA000042, were included for comparison. Grey highlighted PIES were overlooked as edit sites in this transcriptome analysis and identification criteria. [file 1471-2164-15-31-S3.pdf]

Supplemental Table 2. C to U RNA edit sites detected in the tobacco mitochondrial transcriptome alignment.

Edit % = the proportion of aligned nucleotides that differed from the genome sequence. PIES = Previously Identified Edit Sites (Genbank accession BA000042).

Grey highlighted PIES were overlooked as edit sites using our identification criteria.

Gene Name	Genome Position	Edit %	Depth of Coverage	PIES	Gene Name	Genome Position	Edit %	Depth of Coverage	PIES	Gene Name	Genome Position	Edit %	Depth of Coverage	PIES
mat-R	4159	59.80%	>1000	+	ccmC	19300	0.00%	77	+	atp6	25109	20.80%	48	+
	4169	93.20%	>1000	+		19304	32.98%	97	+		25114	25.60%	39	+
	4226	90.00%	>1000	+		19309	8.18%	110	+		25165	98.60%	>1000	+
	4257	96.20%	>1000	+		19316	35.60%	132	+		25222	97.10%	>1000	+
	4279	96.20%	>1000	+		19321	83.10%	338	+		25301	99.30%	>1000	+
	4313	90.90%	>1000	+		19346	86.90%	>1000	+	cob	40964	99.10%	>1000	+
	4334	93.90%	>1000	+		19367	94.00%	>1000	+		41066	99.20%	>1000	+
	5600	89.70%	>1000	+		19368	96.60%	>1000	+		41140	99.50%	>1000	+
	5687	77.20%	>1000	+		19436	94.50%	>1000	+		41195	96.90%	>1000	+
	5777	94.60%	>1000	+		19486	98.40%	>1000	+		41368	97.60%	>1000	+
	5820	90.70%	>1000	+		19583	93.90%	>1000	+		41480	97.50%	>1000	+
nad1_ex4	6710	82.60%	>1000	+		19588	98.50%	>1000	+		41667	>0.10%	>1000	+
	6719	88.80%	>1000	+		19606	95.50%	>1000	+		41690	99.20%	>1000	+
	6740	53.80%	491			19634	96.80%	>1000	+		41723	98.10%	>1000	+
	18704	75.30%	>1000			19652	94.20%	>1000	+		41750	93.70%	>1000	+
	18722	78.70%	>1000			19664	96.60%	>1000	+		41934	44.21%	>1000	+
ccmC	19094	94.10%	>1000	+		19683	2.37%	>1000	+		41995	55.26%	>1000	+
	19111	96.90%	>1000	+		19691	94.40%	>1000	+	rps14	43397	82.60%	>1000	+
	19117	92.30%	>1000	+		19762	88.60%	>1000	+	rpl5	43746	75.60%	>1000	+
	19143	1.86%	537	+	atp6	24620	97.10%	>1000	+		43749	84.20%	>1000	+
	19148	67.30%	388	+		24667	98.00%	>1000	+		44098	93.30%	>1000	+
	19159	56.30%	183	+		24674	99.00%	>1000	+		44166	70.50%	>1000	+
	19161	4.32%	208	+		24682	91.40%	>1000	+		44194	81.30%	>1000	+
	19162	59.00%	205	+		24811	99.90%	>1000	+		44199	92.50%	>1000	+
	19192	95.00%	>1000	+		24853	96.40%	>1000	+		44223	60.80%	>1000	+
	19199	90.20%	>1000	+		24878	97.00%	>1000	+	nad1_ex5	44590	90.20%	>1000	+
	19219	95.60%	>1000			25069	56.30%	71	+		44599	93.10%	>1000	+
	19270	97.90%	>1000	+		25084	68.00%	75	+		44629	96.40%	>1000	+
	19294	72.00%	304	+		25102	40.00%	75	+		44704	86.40%	>1000	+

Gene Name	Genome Position	Edit %	Depth of Coverage	PIES	Gene Name	Genome Position	Edit %	Depth of Coverage	PIES	Gene Name	Genome Position	Edit %	Depth of Coverage	PIES
nad1_ex5	44748	87.80%	>1000	+	orf265a	85532	87.50%	>1000		atp4	114202	85.30%	373	+
	44772	14.57%	926	+		86326	81.20%	>1000			114223	94.40%	>1000	+
	44784	63.73%	923	+		86340	88.50%	>1000	+		114235	95.50%	>1000	+
	44787	62.10%	872	+	nad3	86399	76.90%	>1000	+		114361	97.90%	>1000	+
	44793	55.90%	835	+		86438	92.60%	>1000	+		114379	99.40%	>1000	+
	44816	69.50%	909			86455	41.05%	>1000	+		114391	97.50%	>1000	+
	74362	97.40%	>1000			86456	86.80%	>1000	+	nad4L	114660	98.20%	>1000	+
	74390	96.30%	>1000			86532	44.21%	>1000	+		114744	86.60%	>1000	+
	74443	98.30%	>1000			86540	91.50%	>1000	+		114753	90.00%	>1000	+
	74628	94.40%	465			86602	66.80%	786	+		114762	95.50%	>1000	+
	74677	71.60%	521			86603	71.00%	680	+		114783	81.20%	>1000	+
	74685	89.40%	463			86609	58.40%	586	+		114810	82.70%	>1000	+
orf197a	75164	96.70%	>1000			86624	13.41%	313	+		114831	78.50%	>1000	+
	75191	93.60%	974			86641	5.05%	297	+		114855	36.04%	545	+
	75225	89.30%	403			86645	10.00%	320	+		114886	79.90%	>1000	+
	75231	80.30%	386			86660	73.10%	666	+		114894	82.50%	>1000	+
	75284	95.30%	>1000			86669	79.10%	>1000	+		114900	81.60%	>1000	+
	75340	88.60%	>1000			86711	91.50%	>1000	+		114933	92.50%	>1000	+
	75369	90.00%	>1000			86738	89.90%	>1000	+		115729	69.10%	>1000	
	75499	63.40%	235			86743	91.60%	>1000	+		124295	65.70%	>1000	
nad2_ex1	76596	87.20%	>1000	+	rps12	86903	86.20%	>1000	+	ccmFN	124599	97.00%	>1000	+
sdh3	77451	89.50%	>1000	+		86945	91.10%	>1000	+		124634	93.30%	>1000	+
	77717	57.00%	628			86995	94.00%	>1000	+		124646	94.50%	>1000	+
	77971	55.90%	513			87068	87.20%	>1000	+		124670	98.20%	>1000	+
	77996	56.70%	566			93293	97.80%	>1000			124713	96.70%	>1000	+
	78399	52.80%	267		atp4	114034	96.30%	>1000	+		124731	95.00%	>1000	+
	79436	60.00%	405			114043	95.30%	>1000	+		124764	93.60%	>1000	+
	80509	66.20%	130			114055	97.30%	>1000	+		124782	97.00%	>1000	+
orf265a	85521	94.10%	>1000			114199	52.20%	67	+		124814	97.70%	>1000	+

Gene Name	Genome Position	Edit %	Depth of Coverage	PIES	Gene Name	Genome Position	Edit %	Depth of Coverage	PIES	Gene Name	Genome Position	Edit %	Depth of Coverage	PIES
ccmFN	124842	99.10%	>1000	+	cox1	130046	99.40%	>1000	+	cox1	145810	98.30%	>1000	+
	125160	98.50%	>1000	+		130273	89.10%	>1000			145900	93.60%	>1000	+
	125309	96.30%	>1000	+		130277	98.20%	>1000	+		145902	64.10%	>1000	+
	125324	97.30%	>1000	+		130286	81.10%	>1000			145910	70.80%	89	+
	125336	97.10%	>1000	+	rps10_ex1	130483	95.80%	>1000	+		145942	98.90%	>1000	+
	125396	97.90%	>1000	+		130512	96.80%	>1000	+		145972	98.10%	>1000	+
	125405	91.00%	>1000	+		131329	90.10%	>1000		rps19	152330	85.40%	>1000	+
	125731	87.80%	>1000	+	rps10_ex2	131350	94.80%	>1000	+		152352	39.03%	>1000	+
	125738	96.60%	>1000	+		131545	93.60%	>1000	+		152377	85.90%	>1000	+
	125820	89.10%	>1000	+		131586	95.70%	>1000	+		152378	67.30%	>1000	+
	125847	93.80%	>1000	+		131636	94.60%	>1000	+		152435	87.40%	>1000	+
	125855	96.70%	>1000	+		132471	84.40%	643		rps3_ex2	154079	89.00%	554	+
	125952	95.80%	>1000	+		142436	91.50%	>1000			154113	5.80%	799	+
	125961	96.10%	>1000	+	nad1_ex3	142928	95.40%	>1000	+		154499	90.30%	>1000	+
	125966	92.30%	>1000	+		142983	99.10%	>1000	+		154700	79.10%	>1000	+
	126065	96.20%	>1000	+		143026	84.10%	>1000	+		155018	85.90%	>1000	+
cox1	128789	98.80%	>1000	+		143027	96.70%	>1000	+		155351	96.10%	>1000	+
	128799	98.30%	>1000	+		143063	90.60%	>1000	+		155378	98.50%	>1000	+
	128855	99.50%	>1000	+		143070	97.80%	>1000	+		155542	96.80%	>1000	+
	128883	96.00%	>1000	+		143071	94.40%	>1000	+		155575	97.40%	>1000	+
	129102	87.40%	>1000	+	nad 1_ex2	144339	94.40%	>1000	+		155606	91.70%	>1000	+
	129527	98.90%	>1000	+		144374	96.00%	>1000	+	rps3	155575	97.40%	>1000	+
	129542	70.60%	741	+		144662	84.30%	>1000			155606	91.70%	>1000	+
	129620	20.33%	59	+	rps13	144993	92.20%	>1000	+	rpl16	155754	94.80%	>1000	+
	129698	99.20%	>1000	+		145180	96.50%	>1000	+		155882	96.70%	>1000	+
	129737	95.60%	>1000	+		145224	92.50%	>1000	+		156057	91.20%	>1000	+
	129836	99.10%	>1000	+	atp9	145769	97.80%	>1000	+	cox2 _ex1	156389	93.00%	>1000	+
	130034	96.20%	>1000	+	cox1	145780	98.40%	>1000	+		156422	99.60%	>1000	+

Gene Name	Genome Position	Edit %	Depth of Coverage	PIES	Gene Name	Genome Position	Edit %	Depth of Coverage	PIES	Gene Name	Genome Position	Edit %	Depth of Coverage	PIES
cox2 _ex1	156458	97.00%	>1000	+	nad4_ex1	182535	91.50%	>1000	+	nad4_ex4	190797	81.70%	>1000	+
	156512	99.10%	>1000	+		182566	99.00%	>1000	+		190802	89.70%	>1000	+
	156604	98.90%	>1000	+		182731	95.90%	>1000	+	orf216	191167	76.80%	>1000	+
	156629	82.10%	>1000	+		182745	98.30%	>1000	+		191231	97.90%	>1000	+
	156730	98.00%	>1000	+		182802	94.40%	>1000	+		191250	64.00%	>1000	
cox2_ex2	158154	99.70%	>1000	+		182805	97.20%	>1000	+		191366	92.70%	>1000	+
	158172	97.70%	>1000	+		182806	98.30%	>1000	+		192075	84.40%	>1000	
	158187	97.90%	>1000	+	nad4_ex2	184364	94.40%	>1000	+	nad5_ex1	192303	96.30%	>1000	+
	158268	99.70%	>1000	+		184395	97.30%	>1000	+		193218	95.40%	>1000	
	158343	98.60%	>1000	+		184446	96.70%	>1000	+	nad5_ex2	193234	93.80%	>1000	+
	158432	97.90%	>1000	+		184554	96.00%	>1000	+		193350	97.60%	>1000	+
	158453	98.00%	>1000	+		184623	99.00%	>1000	+		193351	99.30%	>1000	+
nad5_ex3	172744	93.90%	>1000	+		184674	99.10%	>1000	+		193366	97.10%	>1000	+
	172804	95.10%	>1000	+		184739	95.40%	>1000	+		193390	98.60%	>1000	+
	172822	99.20%	>1000	+	nad4_ex3	187902	14.85%	>1000	+		193531	97.10%	>1000	+
	172834	94.10%	>1000	+		187931	98.50%	>1000	+		193590	98.90%	>1000	+
	172864	98.40%	>1000	+		187935	97.70%	>1000	+		193600	90.40%	>1000	+
	174240	97.50%	>1000	+		187941	97.90%	>1000	+		193621	96.70%	>1000	+
nad5_ex4	174261	93.00%	>1000	+		188034	90.50%	>1000	+		193705	99.40%	>1000	+
	174263	92.70%	>1000	+		188054	73.90%	>1000	+		193717	98.90%	>1000	+
	174303	99.40%	>1000	+		188073	90.70%	788	+		193811	94.00%	>1000	+
trnD(guc)	179057	90.90%	408			188076	90.00%	841	+		193827	96.80%	>1000	+
nad4_ex1	182398	92.40%	>1000	+		188097	97.60%	>1000	+		194302	98.60%	>1000	+
	182443	87.20%	>1000	+		188232	97.90%	>1000	+		194441	86.20%	>1000	
	182446	98.60%	>1000	+		188280	97.40%	>1000	+		194708	94.80%	679	
	182476	97.90%	>1000	+		188298	93.10%	>1000	+		194897	94.30%	772	
	182493	81.70%	>1000	+		190759	79.40%	374		nad2_ex3	201212	94.80%	>1000	+
	182523	89.70%	>1000	+	nad4_ex4	190769	25.00%	428	+	nad2_ex4	203737	97.40%	>1000	+
	182527	95.80%	>1000	+		190781	36.00%	821	+		203749	95.60%	>1000	+

Gene Name	Genome Position	Edit %	Depth of Coverage	PIES	Gene Name	Genome Position	Edit %	Depth of Coverage	PIES	Gene Name	Genome Position	Edit %	Depth of Coverage	PIES
nad2_ex4	203758	96.70%	>1000	+	cox3	259157	84.80%	297	+	nad2_ex1	276869	85.20%	>1000	+
	203877	98.10%	>1000	+		259262	97.60%	>1000	+		276999	60.20%	206	
	203907	98.50%	>1000	+		259355	97.20%	>1000	+		278096	87.20%	>1000	
	203911	96.90%	>1000	+		259496	98.70%	>1000	+	sdh3	278951	88.10%	>1000	
	203977	98.60%	>1000	+		259597	99.10%	>1000	+		279217	53.30%	659	
	204195	98.20%	>1000	+	orf125e	259721	55.70%	>1000	+		279471	52.60%	534	
	204196	98.00%	>1000	+		259723	97.90%	>1000	+		279496	55.50%	586	
	204225	97.30%	>1000	+		259760	79.40%	>1000	+		279899	52.80%	271	
nad2_ex5	205707	97.50%	>1000	+		259771	99.70%	>1000	+		280936	55.80%	428	
	205809	90.90%	>1000	+		259827	99.60%	>1000	+		282009	73.70%	133	
	205817	75.50%	580	+		259916	88.30%	>1000		orf265	287021	91.20%	>1000	
	205818	51.70%	572	+		259921	94.30%	>1000			287032	85.80%	>1000	
	205825	5.25%	495	+		260162	52.70%	313			287826	80.00%	>1000	
	205866	92.20%	949	+	atp1	262325	99.20%	>1000	+		287840	87.60%	>1000	
	205989	1.10%	171	+		262464	96.50%	>1000	+	nad3	287899	78.00%	>1000	
	222196	82.30%	>1000			262502	96.40%	>1000	+		287938	93.40%	>1000	
	222840	68.70%	>1000			262578	98.90%	>1000	+		287956	88.50%	>1000	
trnE(uuc)	247026	96.80%	221			262701	99.30%	>1000	+		288040	93.50%	>1000	
	247080	92.80%	469			262776	99.10%	>1000	+		288102	67.10%	797	
orfB/atp8	257766	24.20%	>1000	+		276539	73.50%	494			288103	69.70%	687	
	257783	96.10%	>1000	+	nad2_ex1	276569	95.60%	>1000	+		288109	59.50%	602	
	257794	94.40%	>1000	+		276595	98.50%	>1000	+		288160	71.50%	688	
	257812	92.00%	>1000	+		276664	98.70%	>1000	+		288169	78.20%	>1000	
	257901	55.70%	>1000	+		276691	96.50%	>1000	+		288211	93.40%	>1000	
	258179	98.00%	>1000	+		276725	91.10%	644	+		288238	91.60%	>1000	
	258211	67.80%	>1000	+		276731	79.00%	343	+		288243	91.00%	>1000	
cox3	259088	99.00%	>1000	+		276736	76.00%	175	+	nad1_ex1	288662	95.30%	>1000	+
	259141	58.10%	>1000	+		276751	82.50%	143	+		288712	98.10%	>1000	+
	259147	83.20%	346	+		276784	95.80%	>1000	+		288754	97.80%	>1000	+
	259154	46.55%	232	+		276840	88.00%	>1000	+		288755	98.30%	>1000	+

Gene Name	Genome Position	Edit %	Depth of Coverage	PIES
ccmFc_ex1	307094	83.60%	617	+
	307106	90.80%	403	+
	307108	91.80%	403	+
	307159	92.50%	613	+
	307178	89.80%	502	+
	307202	91.00%	335	+
	307207	80.80%	308	+
	307211	78.60%	351	+
	307366	96.20%	>1000	+
	307390	90.40%	>1000	+
	307447	95.80%	>1000	+
	307462	91.90%	>1000	+
	307762	95.60%	994	+
	307763	97.00%	994	+
	308719	91.40%	304	
ccmFc_ex2	309170	97.10%	>1000	+
	309244	96.60%	>1000	+
	309249	82.20%	>1000	+
	309278	97.80%	>1000	+
nad6	312895	86.50%	>1000	
	313273	96.90%	>1000	+
	313303	97.90%	>1000	
	313361	96.10%	>1000	
	313369	98.60%	>1000	
nad6	313376	89.40%	>1000	
	313438	94.70%	>1000	
	313535	90.10%	>1000	
rps4	314156	94.40%	>1000	+
	314170	95.20%	>1000	+
rps4	314221	60.20%	>1000	+
	314236	90.90%	>1000	+
	314246	47.30%	1410	+
	314257	79.40%	>1000	+
	314680	95.50%	>1000	+
	314688	45.26%	>1000	+
	314728	77.90%	>1000	+
	314763	63.80%	>1000	+
	314827	93.70%	>1000	+
	314872	95.40%	>1000	+
	314884	96.00%	>1000	+
	314896	95.40%	>1000	+
	314998	95.10%	>1000	+
	315124	87.50%	>1000	+
trnH(gug)	332001	67.90%	527	
	332015	62.60%	486	
	334383	54.70%	223	
	336024	94.40%	>1000	
nad9	336202	98.20%	>1000	+
	336277	91.90%	>1000	+
	336408	96.70%	>1000	+
	336438	97.20%	>1000	+
	336478	97.50%	>1000	+
	336508	98.70%	>1000	+
	336549	97.50%	>1000	+
trnW(cca)	337885	95.70%	>1000	
tatC(orfX)	350617	95.60%	>1000	+
	350662	90.30%	>1000	+
	350663	94.60%	>1000	+
tatC(orfX)	350714	94.50%	>1000	+
	350720	85.60%	>1000	+
	350752	87.40%	>1000	+
	350776	87.40%	563	+
	350782	52.50%	547	+
	350789	92.00%	501	+
	350825	91.50%	>1000	+
	350833	92.90%	>1000	+
	350858	97.10%	>1000	+
	350889	40.00%	>1000	+
	350893	91.30%	>1000	+
	350923	85.50%	833	+
	350951	0.59%	338	+
	350984	85.21%	257	+
	350984	84.80%	256	+
	350986	2.67%	262	+
	350999	74.80%	322	+
	351002	65.20%	353	+
	351045	31.33%	>1000	+
	351068	93.60%	>1000	+
	351078	68.42%	>1000	+
	351106	81.80%	802	+
	351107	22.22%	797	+
	351128	84.80%	858	+
	351142	93.70%	589	+
	351152	96.30%	542	+
	351199	82.50%	555	+
	351200	22.88%	555	+
	351202	87.10%	533	+

Gene Name	Genome Position	Edit %	Depth of Coverage	PIES	Gene Name	Genome Position	Edit %	Depth of Coverage	PIES	Gene Name	Genome Position	Edit %	Depth of Coverage	PIES
tatC(orfX)	351218	17.77%	303	+	ccmB	359565	22.05%	136	+	ccmB	359983	95.70%	>1000	+
	351219	11.97%	334	+		359566	38.12%	160	+		360232	95.30%	>1000	
	351230	83.60%	298	+		359658	92.70%	>1000	+	orf159b	360524	96.80%	>1000	
	351266	89.40%	624	+		359676	88.80%	>1000	+		360629	98.00%	>1000	
	351271	30.00%	622	+		359685	95.10%	>1000	+		360630	85.70%	>1000	
	351293	10.59%	387	+		359709	45.08%	>1000	+		360662	96.00%	>1000	
	351304	75.10%	373	+		359710	91.80%	>1000	+	rpl2_ex2	363743	82.40%	642	+
	351305	17.42%	373	+		359739	93.70%	>1000	+	nad7_ex1	375344	99.60%	>1000	+
	351314	70.10%	442	+		359752	85.90%	>1000	+		375386	99.80%	>1000	+
	351701	84.70%	248			359796	96.30%	>1000	+		375407	96.10%	>1000	+
	351896	78.80%	226			359800	96.50%	>1000	+		375422	88.80%	>1000	+
ccmB	359400	90.70%	633	+		359839	65.80%	465	+		375453	98.50%	>1000	+
	359411	16.83%	398	+		359847	50.50%	212	+		375460	96.60%	>1000	+
	359415	84.00%	344	+		359848	85.84%	212	+		375537	97.80%	>1000	+
	359417	34.46%	264	+		359852	9.09%	132	+		375547	61.05%	>1000	+
	359420	8.89%	281	+		359857	0.80%	124	+		375566	97.10%	>1000	+
	359452	91.90%	>1000	+		359866	2.56%	117	+		375584	95.78%	>1000	+
	359459	79.90%	>1000	+		359874	8.16%	98	+		375713	91.80%	625	
	359500	78.50%	354	+		359875	10.30%	97	+	nad7_ex2	377477	98.10%	>1000	+
	359509	76.10%	201	+		359884	37.07%	89	+		377506	95.10%	>1000	+
	359520	8.98%	89	+		359886	16.09%	87	+		377507	95.80%	>1000	+
	359521	0.00%	88	+		359923	82.60%	242	+		377522	99.30%	>1000	+
	359526	6.74%	89	+		359926	62.40%	242	+		377668	99.00%	>1000	+
	359532	15.90%	88	+		359938	23.17%	82	+		377863	93.80%	>1000	+
	359536	9.52%	84	+		359941	14.10%	78	+		377902	98.00%	>1000	+
	359544	16.09%	87	+		359944	26.92%	78	+		377911	99.30%	>1000	+
	359551	7.86%	89	+		359948	36.95%	230	+	nad7_ex2	377930	99.20%	>1000	+
	359555	1.09%	91	+		359968	86.60%	>1000	+		377995	97.80%	>1000	+

Gene Name	Genome Position	Edit %	Depth of Coverage	PIES
nad7_ex2	378002	96.40%	>1000	+
	378042	89.30%	>1000	
nad7_ex3	379492	96.50%	>1000	+
	379501	97.30%	>1000	+
nad7_ex4	380517	33.68%	>1000	+
	380533	91.80%	>1000	+
	380539	91.00%	>1000	+
	380571	65.50%	>1000	+
	380578	92.30%	>1000	+
	424956	99.80%	>1000	
	425112	97.60%	>1000	
	425378	95.50%	491	
	425427	75.80%	566	
	425435	91.60%	501	
orf197b	425914	96.10%	>1000	
	425941	91.50%	>1000	
	425975	88.30%	409	
	425981	77.80%	392	
	426034	95.80%	>1000	
	426090	88.60%	>1000	
	426119	84.40%	>1000	
	426249	63.70%	204	
	427346	87.10%	>1000	
sdh3	428201	86.30%	>1000	
	428467	57.60%	668	
	428721	53.90%	571	
	428746	57.80%	592	

Gene Name	Genome Position	Edit %	Depth of Coverage	PIES
	429149	55.60%	268	
	430186	60.30%	433	
