# Supplementary material for: Deep sequencing of the tobacco mitochondrial transcriptome reveals expressed ORFs and numerous editing sites outside coding regions
Source: BMC Genomics. 2014 Jan 17;15:31. doi: 10.1186/1471-2164-15-31 (PMC3898247; doi:10.1186/1471-2164-15-31)
Supplement: Additional file 4: Table S3 — Summary of edit sites in non-coding regions and location of nucleotide in relation to the mono- and poly-cistronic transcripts. [file 1471-2164-15-31-S4.pdf]

Supplemental Table 3. Summary of Edit Sites in non coding regions

Edit Site	Transcript Region
6740	5' UTR of matR :: nad1_ex4 polycistronic transcript
18704	5' UTR of ccmC transcript
18722	5' UTR of ccmC transcript
44816	5' UTR of cob :: rps14 :: rpl5 :: nad1_ex5 transcript
74362	region with DOC >200 but no identifiable function or reading frame
74390	region with DOC >200 but no identifiable function or reading frame
74443	region with DOC >200 but no identifiable function or reading frame
74628	3' UTR of nad2_ex1 :: sdh3 polycistronic transcript
74677	3' UTR of nad2_ex1 :: sdh3 polycistronic transcript
74685	3' UTR of nad2_ex1 :: sdh3 polycistronic transcript
75340	3' UTR of nad2_ex1 :: sdh3 polycistronic transcript
75369	3' UTR of nad2_ex1 :: sdh3 polycistronic transcript
75499	3' UTR of nad2_ex1 :: sdh3 polycistronic transcript
78399	3' UTR of nad2_ex1 :: sdh3 polycistronic transcript
79436	3' UTR of nad2_ex1 :: sdh3 polycistronic transcript
80509	3' UTR of nad2_ex1 :: sdh3 polycistronic transcript
86326	intergenic region between orf265 and nad3
86340	intergenic region between orf265 and nad3
93293	region with DOC >200 but no identifiable function or reading frame
115729	5' UTR of orf25(atp4)::nad4L polycistronic transcript
124295	3' UTR of ccmFN :: cox1 :: rps10_ex1 :: rps10_ex2 polycistronic transcript
131329	intergenic region between rps10_ex1 and rps10_ex2
131636	5' UTR of ccmFN :: cox1 :: rps10_ex1 :: rps10_ex2 polycistronic transcript
132471	5' UTR of ccmFN :: cox1 :: rps10_ex1 :: rps10_ex2 polycistronic transcript
142436	3' UTR of nad1_ex3 :: nad1_ex2 :: rsp13 :: atp9 polycistronic transcript
144662	intergenic region between nad1_ex2 and rps13
190759	intergenic region between nad4_ex3 and nad4_ex4
192075	intergenic region between nad5_ex1 and orf216

Edit Site	Transcript Region
193218	intergenic region between nad5_ex1 and nad5_ex2
194441	3'UTR of nad4_ex1 :: nad4_ex2 :: nad4_ex3 :: nad4_ex4 :: nad5_ex1 :: nad5_ex2 polycistronic transcript
194708	3'UTR of nad4_ex1 :: nad4_ex2 :: nad4_ex3 :: nad4_ex4 :: nad5_ex1 :: nad5_ex2 polycistronic transcript
194897	3'UTR of nad4_ex1 :: nad4_ex2 :: nad4_ex3 :: nad4_ex4 :: nad5_ex1 :: nad5_ex2 polycistronic transcript
222196	5' UTR of nad5_ex3
222840	5' UTR of nad5_ex3
247080	region with DOC >200 but no identifiable function or reading frame
258211	intergenic region between atp8 and cox3
259916	intergenic region between cox3 and atp1
259921	intergenic region between cox3 and atp1
260162	intergenic region between cox3 and atp1
276539	3' UTR of the sdh3 :: nad2_ex1 :: nad2_ex2 polycistronic transcript created by the junction of Repeat 2 and Sequence 3
276999	intergenic region between nad2_ex2 and nad2_ex1
278096	5' UTR of the sdh3 :: nad2_ex1 :: nad2_ex2 polycistronic transcript created by the junction of Repeat 2 and Sequence 3
279217	5' UTR of the sdh3 :: nad2_ex1 :: nad2_ex2 polycistronic transcript created by the junction of Repeat 2 and Sequence 3
279471	5' UTR of the sdh3 :: nad2_ex1 :: nad2_ex2 polycistronic transcript created by the junction of Repeat 2 and Sequence 3
279496	5' UTR of the sdh3 :: nad2_ex1 :: nad2_ex2 polycistronic transcript created by the junction of Repeat 2 and Sequence 3
279899	5' UTR of the sdh3 :: nad2_ex1 :: nad2_ex2 polycistronic transcript created by the junction of Repeat 2 and Sequence 3
280936	5' UTR of the sdh3 :: nad2_ex1 :: nad2_ex2 polycistronic transcript created by the junction of Repeat 2 and Sequence 3
282009	5' UTR of the sdh3 :: nad2_ex1 :: nad2_ex2 polycistronic transcript created by the junction of Repeat 2 and Sequence 3
287826	intergenic region between orf265b and nad3
287840	intergenic region between orf265b and nad3
308719	intergenic region between ccmFc_ex1 and ccmFc_ex2
313535	intergenic region between nad6 and rps4
334383	5' UTR of nad9
336024	5' UTR of nad9
351701	5' UTR of tatC(orfX)
351896	5' UTR of tatC(orfX)
360232	intergenic region between ccmB and orf159b
375713	intergenic region between nad7_ex1 and nad7_ex2

Edit Site	Transcript Region
378042	intergenic region between nad7_ex2 and nad7_ex3
424956	region with DOC >200 but no identifiable function or reading frame
425112	region with DOC >200 but no identifiable function or reading frame
425378	3' UTR of the orf197 :: nad2_ex1 :: sdh3 :: orf173 polycistronic transcript created by the fusion of Repeats 1 and 2
425427	3' UTR of the orf197 :: nad2_ex1 :: sdh3 :: orf173 polycistronic transcript created by the fusion of Repeats 1 and 2
425435	3' UTR of the orf197 :: nad2_ex1 :: sdh3 :: orf173 polycistronic transcript created by the fusion of Repeats 1 and 2
426090	intergenic region between orf197 and nad2_ex1
426119	intergenic region between orf197 and nad2_ex1
426249	intergenic region between orf197 and nad2_ex1
427346	intergenic region between orf197 and nad2_ex1
428467	5' UTR of the orf197 :: nad2_ex1 :: sdh3 :: orf173 polycistronic transcript created by the fusion of Repeats 1 and 2
428721	5' UTR of the orf197 :: nad2_ex1 :: sdh3 :: orf173 polycistronic transcript created by the fusion of Repeats 1 and 2
428746	5' UTR of the orf197 :: nad2_ex1 :: sdh3 :: orf173 polycistronic transcript created by the fusion of Repeats 1 and 2
429149	5' UTR of the orf197 :: nad2_ex1 :: sdh3 :: orf173 polycistronic transcript created by the fusion of Repeats 1 and 2
430186	5' UTR of the orf197 :: nad2_ex1 :: sdh3 :: orf173 polycistronic transcript created by the fusion of Repeats 1 and 2
