# Supplementary material for: Deep sequencing of the tobacco mitochondrial transcriptome reveals expressed ORFs and numerous editing sites outside coding regions
Source: BMC Genomics. 2014 Jan 17;15:31. doi: 10.1186/1471-2164-15-31 (PMC3898247; doi:10.1186/1471-2164-15-31)
Supplement: Additional file 5: Table S4 — ORF Average Transcript Abundance and Standard Error (S.E.) as Measured by qRT-PCR and Mann–Whitney Pair-Wise Statistical Analysis. [file 1471-2164-15-31-S5.pdf]

Supplemental Table 4 - Tobacco mitogenome ORF Transcript Abundance as Measured by qRT-PCR

	Leaves				Roots				Flowers			
Transcript	Average Transcript Abundance	S.E.	Mann-Whitney ORF vs. background	Higher Than Background?	Average Transcript Abundance	S.E.	Mann-Whitney ORF vs. background	Higher Than Background?	Average Transcript Abundance	S.E.	Mann-Whitney ORF vs. background	Higher Than Background?
background	4.2	0.8	n/a	n/a	23.8	11.2	n/a	n/a	231.4	31.3	n/a	n/a
cox2	8767.4	82.1	0.002	Yes	37510.6	51.0	0.002	Yes	3091.5	2.4	0.002	yes
orf177	113.5	22.5	0.002	Yes	1600.1	230.5	0.002	Yes	481.7	118.3	0.017	yes
orf197	33.1	10.3	0.1	Yes	44.9	10.8	0.1	Yes	791.8	171.0	0.002	yes
orf265/atp8	15243.0	1286.1	0.002	Yes	12939.4	2601.7	0.002	Yes	2965.7	343.9	0.002	yes
orf129	80.3	9.3	0.002	Yes	1019.0	202.4	0.002	Yes	255.1	16.6	0.485	no
orf175	14.5	1.2	0.002	Yes	148.2	37.0	0.002	Yes	5.2	1.5	0.002	no
orf25/atp4	4649.0	462.4	0.1	Yes	11081.9	1063.2	0.002	Yes	1928.9	263.8	0.002	yes
orf222	1803.7	667.5	0.1	Yes	2391.6	255.7	0.1	Yes	979.8	181.1	0.009	yes
orf239	53.4	14.7	0.1	Yes	191.8	17.9	0.1	Yes	687.3	101.4	0.002	yes
orf216	3080.7	925.2	0.1	Yes	7229.2	1288.1	0.1	Yes	1869.2	237.5	0.002	yes
orf306	37.4	9.4	0.002	Yes	146.9	31.5	0.002	Yes	8.0	1.6	0.002	no
orf147	73.8	13.4	0.1	Yes	262.3	35.6	0.1	Yes	1365.9	222.5	0.002	yes
orf144	24.4	4.1	0.002	Yes	132.0	23.0	0.002	Yes	25.8	4.5	0.002	no
orf118	153.1	10.9	0.002	Yes	1067.8	148.0	0.002	Yes	4.3	1.4	0.002	no
orf160	864.3	37.6	0.002	Yes	3804.0	234.1	0.002	Yes	1123.0	123.7	0.002	yes
orf125d	11.5	1.6	0.002	Yes	99.0	31.5	0.002	Yes	112.9	22.1	0.048	no
orf115	361.5	87.0	0.002	Yes	1393.2	214.8	0.002	Yes	356.1	97.9	0.24	no
orf166b	360.7	20.3	0.002	Yes	2619.4	348.4	0.002	Yes	436.2	163.4	0.31	no
orf159/rpl10	3360.0	611.7	0.1	Yes	10275.6	521.7	0.1	Yes	2254.5	294.3	0.002	yes
