# Supplementary material for: Deep sequencing of the tobacco mitochondrial transcriptome reveals expressed ORFs and numerous editing sites outside coding regions
Source: BMC Genomics. 2014 Jan 17;15:31. doi: 10.1186/1471-2164-15-31 (PMC3898247; doi:10.1186/1471-2164-15-31)
Supplement: Additional file 6: Table S5 — ORF Average Transcript Abundance and Standard Error (S.E.) in the Supernatant and Pellet Portions of Polysome Analyses as Measured by qRT-PCR and Mann–Whitney Pair-Wise Statistical Analysis. [file 1471-2164-15-31-S6.pdf]

Supplemental Table 5 - Tobacco Mitogenome ORF Polysome Association as Measured by qRT-PCR

Transcript	Super copy #	S.E.	Super M-W test	Pellet copy #	S.E.	Pellet M-W test
**Cox2**	24757	181.8	0.002	10856	8.9	0.002
**Cox2 +EDTA**	22734	3856.6	n/a	Undetectable	n/a	n/a
**Background**	20	5.6	n/a	134	32.9	n/a
**Orf177**	212	40.9	0.002	648	49.3	0.002
**Orf129b**	147	45.3	0.004	289	52.1	0.002
**Orf175**	52	17.9	0.132	0	0.1	0.31
**Orf306**	56	11.5	0.026	1	0.5	0.002
**Orf144**	64	12.2	0.026	4	1.6	0.009
**Orf118**	340	68.3	0.002	0	0.1	0.002
**Orf160**	736	118.8	0.002	2117	132.8	0.002
**Orf125d**	115	36.0	0.002	60	18.4	0.065
**Orf115**	507	127.4	0.002	338	49.9	0.009
**Orf166b**	257	35.7	0.002	1003	99.9	0.002
**Orf25/atp4**	11486	4277.8	0.002	3803	905.7	0.002
**Orf222**	6809	2946.5	0.002	2132	402.4	0.002
**Orf216**	10482	4255.5	0.002	14845	4174.5	0.002
**Orf265/atp8**	41028	18008.5	0.002	19692	5313.9	0.002
**Orf159/rpl10**	19655	8617.9	0.002	5407	882.2	0.002
**Orf197**	1818	709.9	0.002	231	66.3	0.589
**Orf239**	2108	872.4	0.002	206	78.7	0.937
**Orf147**	2606	1043.0	0.002	346	56.7	0.004
