# Supplementary material for: Deep sequencing of the tobacco mitochondrial transcriptome reveals expressed ORFs and numerous editing sites outside coding regions
Source: BMC Genomics. 2014 Jan 17;15:31. doi: 10.1186/1471-2164-15-31 (PMC3898247; doi:10.1186/1471-2164-15-31)
Supplement: Additional file 7: Table S6 — Primers used in this study. [file 1471-2164-15-31-S7.pdf]

Supplemental Table 6. Primers pairs used in qRT-PCR, their optimal annealing ranges, PCR efficiencies, and size of amplicon produced for each pair.

Gene	Left Primer	Right Primer	Anneal	Efficiency	Amplicon
Orf133	tcctctggcattacgacct	caaaacttctaccccgagca	53.0-62.5	99.5	118
Orf25/atp4	acaaattcgcaagctgatcc	cagcaattccccaatccta	53.0-62.5	100	83
Orf222	tcaatcaggggggctaatctg	cgaaagagccttggatcaac	53.0-62.5	98.7	84
Orf216	gttcttggtggatgcaggac	agatccaggaatcccacctt	53.0-62.5	100	114
Orf265/atp8	agcgaaattggaaatgcaac	ggcttccctgtcgagaa	53.0-62.5	100	95
Orf159b	caacctgtccgatgcttctt	aaccctatttcgcccaagtt	53.0-62.5	100	121
Orf197	gaattccccaatcccagagt	tcctactcgcggtatgc	53.0-62.5	103.6	141
Orf265/atp8	ctcacccagggagtacgaaa	aaggtgcaccctcagtatgg	53.0-62.5	100	169
Orf 239	ggtcgcactttatggcattt	tgcgcagtctaccgtttc	53.0-62.5	98.5	144
Orf147	cgcattctaggcacagatc	gggatctcttttctgcaacg	53.0-62.5	106	96
Orf177	gaggccacaagtcaacaac	ggttcccctgtgtcatcaa	53.0-62.5	99	99
Orf129b	gccttggtacggcctataca	gtactgcaacggttgggtc	51.0-65.0	97	88
Orf151	ccccctgaaaagtatcacga	ccaaagcatctatgggttgaa	51.0-62.5	99	124
Orf175	cgctgatcgtggataagac	cttcatcccggattcttcat	55.9-62.5	105	129
Orf134	gtgcggtttctgggaattta	cccctccaacaagaaaagg	50.0-59.5	100	98
Orf306	gcaagcacggttaagggata	tttgccgtccacaaaagaat	53.0-62.5	100	140
Orf138c	ttctccccttaggaccgact	tacagaagccttcgccaact	53.0-62.5	98	116
Orf144	tcgaatcggaacctttatgc	agctatcaatccccgctt	53.0-62.5	100	143
Orf118	gaagcggggattgatagctt	cctatgccagcccaaactaa	51.0-64.1	104	102
Orf160	gggttcatctctctcgacc	ccgccatagagaagagatcg	51.0-64.1	100	80
Orf101d	gtttatccgggggagagatt	gaacccaattcctacggtga	53.0-62.5	100	119
Orf111c	ggcaggcaggcctatatttc	cacgtgaggggttattgctt	53.0-62.5	104	127
Orf125d	aggctgttatgggagacg	tcgaacaccccccttaaaaga	55.9-62.5	102	84
Orfb	cgtcgacttcttgggaaaaa	tctttccattcctcgtgagc	53.0-62.5	98	82
Orf115	gaagtgcagcttgattgtcg	cccttgaacaaaaagccaat	50.0-59.5	96	100
Orf166b	tctggggtggtgcttctatc	agtgtttttgccccttatcg	53.0-62.5	104	81
BKGRND	gttgcaagtcttccgacgat	gaaagagttaagcgcctcca	53.0-62.5	102	146
POSCTRL	gatctcaagacgcagcaaca	gcgacccaagatccatga	51.0-65.0	100	108
